# Life tables for global surveillance of cancer survival (the CONCORD programme): data sources and methods

**DOI:** 10.1186/s12885-017-3117-8

**Published:** 2017-02-27

**Authors:** Devon Spika, Finian Bannon, Audrey Bonaventure, Laura M Woods, Rhea Harewood, Helena Carreira, Michel P Coleman, Claudia Allemani

**Affiliations:** 1 Cancer Survival Group, Department of Non-Communicable Disease Epidemiology, London School of Hygiene and Tropical Medicine, Keppel Street, London, WC1E 7HT UK; 2 Centre for Public Health, Queens University Belfast, Institute of Clinical Sciences, Block B, Grosvenor Road, Belfast, BT12 6BA UK

**Keywords:** Life tables, Mortality, Life expectancy, Generalised linear model, Cubic splines, Global health, Net survival

## Abstract

**Background:**

We set out to estimate net survival trends for 10 common cancers in 279 cancer registry populations in 67 countries around the world, as part of the CONCORD-2 study. Net survival can be interpreted as the proportion of cancer patients who survive up to a given time, after eliminating the impact of mortality from other causes (background mortality). Background mortality varies widely between populations and over time. It was therefore necessary to construct robust life tables that accurately reflected the background mortality in each of the registry populations.

**Methods:**

Life tables of all-cause mortality rates by single year of age and sex were constructed by calendar year for each population and, when possible, by racial or ethnic sub-groups. We used three different approaches, based on the type of mortality data available from each registry. With death and population counts, we adopted a flexible multivariable modelling approach. With unsmoothed mortality rates, we used the Ewbank relational method. Where no data were available from the registry or a national statistical office, we used the abridged UN Population Division life tables and interpolated these using the Elandt-Johnson method. We also investigated the impact of using state- and race-specific life tables versus national race-specific life tables on estimates of net survival from four adult cancers in the United States (US).

**Results:**

We constructed 6,514 life tables covering 327 populations. Wide variations in life expectancy at birth and mortality by age were observed, even within countries. During 1995–99, life expectancy was lowest in Nigeria and highest in Japan, ranging from 47 to 84 years among females and 46 to 78 years among males. During 2005–09, life expectancy was lowest in Lesotho and again highest in Japan, ranging from 45 to 86 years among females and 45 to 80 years among males. For the US, estimates of net survival differed by up to 4% if background mortality was fully controlled with state- and race-specific life tables, rather than with national race-specific life tables.

**Conclusions:**

Background mortality varies worldwide. This emphasises the importance of using population-specific life tables for geographic and international comparisons of net survival.

**Electronic supplementary material:**

The online version of this article (doi:10.1186/s12885-017-3117-8) contains supplementary material, which is available to authorized users.

## Background

The CONCORD-2 study was designed to establish long-term surveillance of cancer survival worldwide, by central analysis of population-based cancer registry data. Net survival from 10 common malignancies was estimated from individual patient data submitted by 279 cancer registries in 67 countries [1].

Net survival of a cohort of cancer patients is estimated as the probability of survival derived solely from the cancer-specific hazard of death. It can be interpreted as the proportion of cancer patients who survive up to a given time after diagnosis (e.g. 5 years), after eliminating the impact of other causes of death (background mortality). This is done by separating the excess hazard of death due to cancer from the background mortality. Background mortality often differs widely between populations, and can even differ substantially within registry populations, for instance by race [2], ethnic group [3] or socio-economic status [4].

Information on background mortality in a given population is obtained from life tables, which are tables of age- and sex-specific death rates or probabilities in a given population at a given point in time. Net survival will be more accurate if the estimates of background mortality are as close as possible to each particular individual’s “real” expected mortality from all causes. Previous international studies of cancer survival [5], including the first CONCORD study [2, 6], have recommended that life tables specific to the area in which cancer patients live should be used, rather than national life tables, which may not account for sub-national differences in mortality. Ideally, these life tables should be by single calendar year, single year of age, sex, and race (or ethnicity) or deprivation when the relevant data are available. Such life tables are, however, not generally available: national statistical offices often only produce life tables for the whole country or major geographic regions.

In this article, we outline the methods used to construct life tables for the CONCORD-2 study, which is the largest comparison of worldwide trends in population-based cancer survival to date. We document the wide variations in life expectancy and age-specific mortality between and within the populations covered by the 279 participating cancer registries. We examine trends in life expectancy in regions within continents, and even within countries. We also investigate the importance of using regional vs. national life tables in the estimation of net survival, by comparing estimates for four adult cancers (breast, colon, lung, prostate) in 44 US registries, using either a US national, race-specific life table [7] or the race- and state-specific life tables that were constructed for the CONCORD-2 study.

## Methods

All 279 cancer registries participating in the CONCORD-2 study were invited to contribute data for patients diagnosed during all or part of the calendar period 1995–2009, with follow-up to 31 December 2009, or a later year. To enable estimation of net survival for these patients, registries were asked to provide data on background mortality for each calendar year for which they submitted cancer data, from the first year of incidence to the last year of follow-up. They were offered the option of supplying their own life tables or providing death and population counts from which we could construct the life tables required.

Some registries also supplied life table data for racial or ethnic sub-populations within their territory: in all, we received data for 327 populations. The Israel National Cancer Registry and all 44 participating United States (US) cancer registries submitted death and population data from which to construct life tables by ethnicity (Israel, national-level) or race (US, state-level). The New Zealand Cancer Registry and the Penang Cancer Registry (Malaysia) provided mortality rates by ethnicity at a national level. Both the Polish National Cancer Registry and the Austrian Cancer Registry submitted mortality rates for the sub-regions covered by their registries (voivodeships for Poland, bundeslands for Austria). Neither registry submitted data by ethnicity.

We classified the data we received into four categories on the basis of their structure and quality: i) death and population counts by single year of age; ii) death and population counts by age group (typically five years); iii) mortality rates by single year of age; and iv) mortality rates by age group. A fifth category included registries from which life table data were unavailable or deemed unreliable. The methods used to construct life tables were different for each of the five categories (Table 1).

**Table 1:** Methods used to construct the CONCORD-2 life tables, by type of data obtained

Data category	Method	No. of registries
Death and population counts		172
i) By single year of age	Multivariable flexible model	72
ii) By age group	Multivariable flexible model	100
Mortality rates		83
iii) By single year of age		73
Smoothed	Calendar year interpolation if necessary	56
Unsmoothed	Ewbank relational model with four parameters	17
iv) By age group	Ewbank relational model with three parameters	10
No reliable life table data available from registry		24
Abridged life table available from the UN Population Division (UNPD)	Abridged UNPD life tables interpolated using Elandt-Johnson method	23
Abridged life table not available from UNPD (Gibraltar only)	Smoothed England life table used	1
		Total: 279

Some registries did not provide life tables (or the corresponding death and population counts) for each calendar year covered by their cancer data. We constructed life tables for any intervening years by linear interpolation of the age-specific death rates. If the calendar span of life tables was shorter than the calendar span of the cancer incidence and follow-up data, life tables for the earliest or latest available year were used for the missing years, i.e. without extrapolation, so that we would have estimates of background mortality for every year included in the cancer data.

### Life tables from death and population counts (categories i and ii)

In all, 172 (62%) of the participating registries provided data on the numbers of deaths and the population size (death and population counts) by age and sex (table 1). A flexible multivariable model (flexible Poisson model) [8] was used to derive the required age- and sex-specific mortality rates. This method was chosen because it was recently recommended for the estimation of smoothed age-specific mortality rates for small populations [8]. This approach also allowed for the modelling of mortality rates by race or ethnicity, where the data were available.

The death counts were modelled separately for each sex and calendar year, within the generalised linear model framework, using a Poisson error and log link. Person-years at risk were used as the offset:

$$ log\left({d}_x\right)={\beta}_0+ f(x)+ log\left( pyr{s}_x\right) $$

where *x* denotes age in years, *d*_*x*_ denotes the age-specific death count, *β*_0_ denotes the coefficient at baseline (i.e. the log of the mortality rate at the reference age), *f*(*x*) denotes a restricted cubic spline function on age, and *pyrs*_*x*_ denotes the age-specific person-years at risk.

The model was implemented using the Stata command *mvrs* (multivariable regression splines) [9] in Stata 13. Splines are made up of piecewise polynomial functions joined at locations called knots. The process we used to select the knot locations is summarised in Additional file 1 and in detail elsewhere [8]. We used the flexible Poisson model with a continuous interaction between race/ethnicity and age to construct race/ethnicity-specific life tables for the Israel National Cancer Registry (ethnicity) and the 44 US states (race). Further details are provided in Additional file 2.

We used three calendar years of death and population counts around a central year, so that the resulting life tables would not be as susceptible to year - on - year fluctuations.

### Life tables from mortality rates (categories iii and iv)

We obtained age-specific mortality rates from 83 (30%) of the participating registries (Table 1). Of these, 73 (88%) provided mortality rates by single year of age (complete life tables) and 10 (12%) provided rates by five-year age group (abridged life tables). Of those registries that submitted complete life tables, 56 (77%) provided smoothed versions for each calendar year submitted (where the raw, age-specific mortality rates had been modelled up to age 99 years to remove any random fluctuations by age) and 17 (23%) did not.

Where the mortality rates we received had not been smoothed, we used the Ewbank relational method [10] to derive a smoothed mortality profile for the given population. The Ewbank method is an extension of the Brass relational method [11]. The Brass method involves plotting the linear relationship between the logits of two survivorship functions, one from a standard life table and the other from observed data. Plotting this linear relationship provides information on two parameters, one for the level of mortality in the model (*a*) and another for the slope of the observed survivorship curve relative to the standard curve, i.e. the relation between young and old age mortality in the observed data relative to the standard (β). These two parameters are then used to determine the shape of a smoothed survivorship function for the observed data. The Ewbank method includes two additional parameters: one for childhood mortality (κ) and another for mortality at older ages (λ). The parameter for childhood mortality applies before the median age at death in the population. The parameter for mortality at older ages applies after the median age at death.

If mortality rates were available by single year of age up to 99 years, we used all four parameters (level of mortality, relation between young and old age mortality, childhood mortality, older-age mortality). In many populations, the median age at death was close to 80 years of age, or higher. For abridged life tables, in which the highest age group is typically for ages 85 years and above, this meant that data to estimate values for the older-age mortality parameter (which only applies after the median age at death) were often available for only one or two age groups. This has previously been found to cause instability in the estimated older-age mortality parameter, leading to unreliable estimates of older-age mortality [12]. For abridged mortality rates, we therefore used only three parameters and constrained the parameter for older-age mortality to be a factor of the parameter for the level of mortality [10].

### Registries for which no reliable data were available (category v)

We were unable to obtain reliable life table data from 24 (8%) registries (Table 1). For 23 of these registries, we used country-level life tables by age group (abridged) for calendar periods 1995–2000, 2000–2005 and 2005–2010 obtained from the UN Population Division (UNPD) [13]. We centred these on years 1997, 2002 and 2007 and smoothed the abridged values using the Elandt-Johnson method [14]. The Elandt-Johnson method has been recommended for deriving single-year-of-age life tables from abridged ones [15]. As above, we produced life tables for individual calendar years by age-specific linear interpolation between the life tables for each of the three calendar periods. For one of these registries, Gibraltar, no life table data were available from the UNPD [13], WHO [16], Global Burden of Disease Study [17] or the Human Mortality Database [18], so we used the life table we constructed for England.

### Evaluation and comparison of derived life tables

Life expectancy at birth is a summary measure of age-specific mortality. We calculated life expectancy at birth, the infant mortality rate (probability of dying between birth and exact age 1), childhood mortality rate (probability of dying between birth and exact age 5), and the probabilities of dying between exact ages 15 and 60, 60 and 85, and 85 and 99 years from each of the derived life tables.

Life expectancies at birth and the probabilities of death were summarised in a standardised report for each cancer registry (see example in Additional file 3). The reports included plots of the smoothed mortality curves on both logarithmic and arithmetic scales.

Performance of the flexible Poisson model was also evaluated from plots of the deviance residuals at each age. Deviance residuals are a measure of how closely the modelled values fit the observed data. The residuals should be approximately normally distributed, with a constant range, if the model fits the data well [19]. We deemed the model to be performing well if the standardised deviance residuals were in the range −2 to +2.

## Results

In total, 6,514 life tables were constructed as part of the CONCORD-2 study: of these, 6,392 life tables were constructed for 223 (80%) registries with the flexible Poisson model, the Ewbank method or the Elandt-Johnson method. A further 35 registries (12.5%) provided smoothed life tables that did not cover all calendar years; for these registries, we constructed 122 life tables by linear interpolation. We received smoothed, complete, life tables for all calendar years from 21 registries (7.5%). No modifications were required for these life tables.

The type of data received varied by continent (Table 2). With the exception of Mauritius, no African registries provided reliable life table data, whereas the great majority of registries from the Americas (North, Central and South) provided death and population counts.

**Table 2:** Type of life table data obtained from each cancer registry: number of registries, by continent

	Type of life table data										
	Mortality rates				Death and population counts				No data		Total
	By single year of age		By age group		By single year of age		By age group				
Continent	No.	(%)	No.	(%)	No.	(%)	No.	(%)	No.	(%)	No.
Africa	–	–	–	–	1	(10)	–	–	9	(90)	10
America (Central and South)	1	(4)	–	–	4	(15)	19	(70)	3	(11)	27
America (North)	–	–	–	–	9	(16)	48	(84)	–	–	57
Asia	3	(6)	6	(12)	11	(22)	19	(38)	11	(22)	50
Europe	63	(49)	3	(2)	47	(37)	14	(11)	1	(1)	128
Oceania	6	(86)	1	(14)	–	–	–	–	–	–	7
Total	73	(26)	10	(4)	72	(26)	100	(36)	24	(9)	279

Life expectancy at birth was higher among females than males in all populations except Mali (Bamako Cancer Registry), throughout 1995–1999, 2000–2004 and 2005–2009 (Additional file 4).

**Fig. 1:**
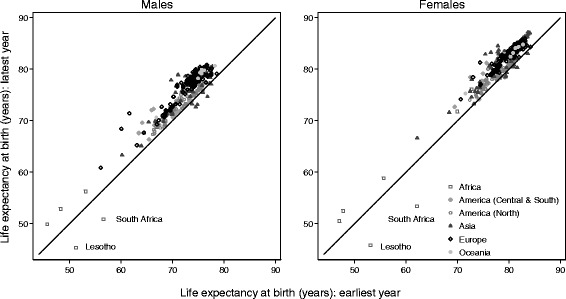
Life expectancy at birth (years), by sex: earliest and latest years of life table data. Each data point represents a single population, either for a registry territory, or for a racial/ethnic sub-population within a given territory. This figure shows the change in life expectancy at birth, by sex, between the earliest and latest years for which life table data were submitted, in the general population of 279 participating registries, covering 327 populations. The diagonal represents ‘no change’ between the first and last years: data points above the diagonal denote an increase in life expectancy for that population

**Fig. 2:**
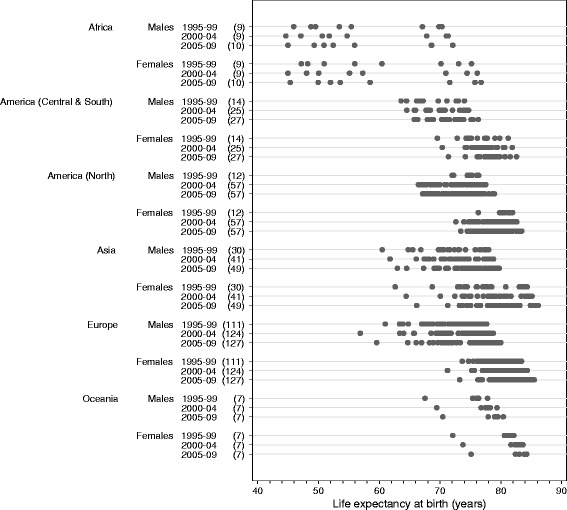
Life expectancy at birth (years): range, by continent, calendar period and sex. The numbers in brackets beside each calendar period denote the number of registries contributing life table data for that calendar period. Each dot on the graph represents a registry population or sub-population

Global variation in life expectancy at birth was very wide (Table 3; Figs. 1 and 2; Additional file 4). Life expectancy was highest in Japan for males and females throughout the 15-year period 1995–2009. It was lowest in Nigeria during 1995–1999 (47 years for females, 46 years for males) and Lesotho during 2000–2004 and 2005–2009 (e.g. 45 years for females, 45 years for males in 2005–2009; Table 3, Additional file 4). During 1995–1999, life expectancy at birth ranged from 47 to 84 years among females and 46 to 78 years among males. During 2005–2009, it ranged from 45 to 86 years among females and 45 to 80 years among males. The largest range within any continent was observed in Africa, where life expectancy in 2005–2009 varied by as much as 30 years between populations (from 45 to 77 years among females, and 45 to 72 years among males). If North Africa is considered separately from East, West and South Africa, however, the ranges within these two regions are narrow. The narrowest range was observed in North America during 1995–1999 (6 years for females, 4 years for males) and Oceania in 2005–2009 (9 years for females, 10 years for males).

**Table 3:** Adult probabilities of dying, and life expectancy at birth: range, by calendar period and sex

		Probability (%) of dying between exact ages:																							
		15 and 60 years						60 and 85 years						85 and 99 years						Life expectancy at birth (years)					
		1995–99			2005–09			1995–99			2005–09			1995–99			2005–09			1995–99			2005–09		
Continent		Lowest	Highest	Range	Lowest	Highest	Range	Lowest	Highest	Range	Lowest	Highest	Range	Lowest	Highest	Range	Lowest	Highest	Range	Lowest	Highest	Range	Lowest	Highest	Range
AFRICA																									
All areas																									
Males		14.5	54.1	39.6	12.9	65.8	52.9	80.7	94.3	13.7	78.7	93.9	15.2	98.5	100.0	1.4	98.0	100.0	1.9	45.9	70.3	24.4	45.0	72.1	27.2
Females		8.7	52.2	43.4	7.6	66.2	58.6	67.0	93.7	26.7	63.8	93.5	29.7	98.2	100.0	1.8	95.2	100.0	4.8	47.2	75.2	28.0	45.4	76.8	31.4
East, West and South																									
Males		32.0	54.1	22.1	21.4	65.8	44.4	88.2	94.3	6.1	80.0	93.9	14.0	98.5	100.0	1.4	98.0	100.0	1.9	45.9	55.4	9.5	45.0	68.6	23.7
Females		27.8	52.2	24.4	10.5	66.2	55.7	77.0	93.7	16.7	63.8	93.5	29.7	98.2	100.0	1.8	95.2	100.0	4.8	47.2	60.4	13.2	45.4	75.7	30.4
North																									
Males		14.5	18.3	3.8	12.9	17.1	4.2	80.7	82.9	2.3	78.7	82.1	3.4	99.5	99.7	0.3	99.4	99.6	0.2	67.1	70.3	3.2	68.6	72.1	3.6
Females		8.7	13.9	5.2	7.6	12.9	5.3	67.0	77.1	10.1	64.6	75.9	11.3	99.0	99.7	0.7	98.9	99.6	0.7	70.2	75.2	5.0	71.6	76.8	5.1
AMERICA (CENTRAL AND SOUTH)																									
All areas																									
Males		13.8	27.5	13.7	12.2	25.4	13.2	55.9	79.6	23.7	51.4	79.9	28.5	81.9	99.4	17.5	87.4	99.7	12.2	63.6	74.0	10.4	65.8	76.3	10.5
Females		6.7	14.0	7.3	5.8	12.6	6.8	45.2	66.6	21.5	41.5	66.2	24.8	82.8	99.1	16.3	85.2	99.1	13.9	69.6	81.2	11.7	71.4	82.6	11.2
AMERICA (NORTH)																									
All areas																									
Males		10.5	17.0	6.5	8.5	26.3	17.8	62.9	77.4	14.5	54.4	76.9	22.5	92.2	99.5	7.3	92.0	99.1	7.1	72.0	76.3	4.4	67.1	78.9	11.8
Females		6.0	10.3	4.4	5.2	15.3	10.1	45.3	60.8	15.5	38.1	70.0	31.9	79.6	92.9	13.3	81.6	98.4	16.8	76.3	81.9	5.6	73.4	83.4	10.0
ASIA																									
All areas																									
Males		7.9	30.7	22.8	7.1	31.7	24.6	60.8	89.2	28.4	55.2	88.2	33.0	91.9	99.6	7.7	88.5	99.8	11.3	60.5	78.0	17.5	63.0	79.7	16.7
Females		4.4	20.8	16.4	3.7	17.5	13.8	37.9	80.8	42.9	31.5	78.9	47.4	87.0	99.6	12.6	83.5	99.6	16.1	62.6	84.3	21.7	66.2	86.2	20.0
East																									
Males		8.6	19.8	11.2	7.3	31.7	24.5	60.8	89.2	28.4	55.2	88.2	33.0	93.6	99.5	5.9	92.0	99.8	7.9	70.6	78.0	7.4	64.5	79.7	15.2
Females		4.4	11.1	6.7	3.7	15.1	11.4	37.9	70.5	32.6	31.5	78.9	47.4	87.0	97.6	10.6	83.5	99.4	15.9	76.0	84.3	8.3	73.2	86.2	12.9
South																									
Males		17.0	30.7	13.7	14.5	27.3	12.7	68.2	85.9	17.7	65.1	83.9	18.8	91.9	99.6	7.7	88.5	99.6	11.1	60.5	73.2	12.7	63.0	75.1	12.1
Females		8.2	20.8	12.7	6.6	17.5	10.9	62.4	80.8	18.4	56.1	77.6	21.5	96.0	99.6	3.6	92.3	99.6	7.3	62.6	77.7	15.1	66.2	80.2	14.0
West																									
Males		7.9	15.3	7.4	7.1	13.8	6.7	65.5	81.3	15.8	58.2	79.6	21.4	93.1	99.6	6.5	94.2	99.6	5.4	70.1	76.7	6.6	71.4	79.1	7.7
Females		5.6	11.8	6.2	4.4	10.4	5.9	53.8	74.1	20.4	46.3	71.3	25.0	92.4	99.5	7.2	87.3	99.4	12.1	72.9	80.8	7.9	74.4	82.8	8.4
EUROPE																									
All areas																									
Males		9.4	41.2	31.9	6.8	45.4	38.6	58.0	87.5	29.4	52.1	90.1	38.0	95.2	99.5	4.3	91.8	99.2	7.4	61.0	77.7	16.7	59.6	80.0	20.4
Females		4.5	14.9	10.4	3.5	15.5	12.0	38.2	72.6	34.4	31.7	71.8	40.1	90.1	99.5	9.5	87.3	99.3	12.0	73.6	83.4	9.8	73.2	85.5	12.3
East																									
Males		18.4	34.9	16.5	14.5	45.4	30.9	75.4	87.3	12.0	70.8	90.1	19.3	96.5	99.5	3.0	95.2	99.0	3.9	63.4	70.7	7.3	59.6	73.3	13.8
Females		7.3	12.2	4.9	5.8	15.5	9.7	59.7	72.6	12.9	49.9	71.8	21.9	96.3	99.5	3.2	93.3	99.3	6.1	74.7	78.5	3.9	73.2	80.9	7.7
North																									
Males		9.4	41.2	31.8	7.6	31.4	23.8	66.4	87.5	21.1	57.7	81.9	24.2	96.1	98.9	2.9	94.4	98.2	3.8	61.0	76.7	15.7	66.1	79.2	13.1
Females		5.7	14.9	9.3	4.5	11.1	6.6	48.7	68.1	19.4	43.9	61.2	17.4	92.9	98.9	6.0	90.4	97.5	7.1	73.6	81.7	8.1	77.0	83.0	6.0
South																									
Males		9.4	19.8	10.5	6.8	17.2	10.3	58.0	83.8	25.8	54.0	78.5	24.5	96.4	98.9	2.5	94.0	99.2	5.2	69.6	77.7	8.1	71.8	80.0	8.2
Females		4.5	8.0	3.4	3.5	6.5	3.0	40.7	67.1	26.4	34.2	60.1	25.9	92.3	98.3	6.0	90.8	98.0	7.2	77.0	83.4	6.4	79.0	85.5	6.5
West																									
Males		9.9	18.6	8.7	7.5	14.9	7.5	59.8	75.6	15.8	52.1	67.6	15.5	95.2	98.7	3.5	91.8	98.3	6.4	72.1	77.1	5.0	75.2	79.9	4.7
Females		4.9	8.1	3.2	4.1	6.9	2.8	38.2	56.1	17.9	31.7	49.6	17.9	90.1	98.3	8.3	87.3	96.6	9.3	79.5	83.3	3.8	81.3	85.2	3.8
OCEANIA																									
All areas																									
Males		8.5	23.8	15.3	7.0	19.6	12.6	62.6	84.5	22.0	52.3	78.1	25.9	92.0	98.7	6.7	89.0	97.2	8.2	67.5	77.8	10.3	70.5	80.4	10.0
Females		5.3	16.7	11.4	4.1	12.7	8.5	46.3	73.8	27.5	38.0	67.0	29.1	86.9	96.4	9.5	83.3	94.9	11.6	72.1	82.1	10.0	75.1	84.2	9.1

Striking changes occurred in life expectancy in some countries between the earliest and latest calendar years for which we obtained life table data (Fig. 1). Among males, life expectancy fell by up to six years in South Africa and Lesotho. Among females, it fell by nine and seven years, respectively. By contrast, life expectancy rose by six years or more in Estonia, Latvia (males only), South Korea, São Paulo (Brazil, males only), Changle (China, females only), Haining (China), and East Germany (males only).

**Fig. 3:**
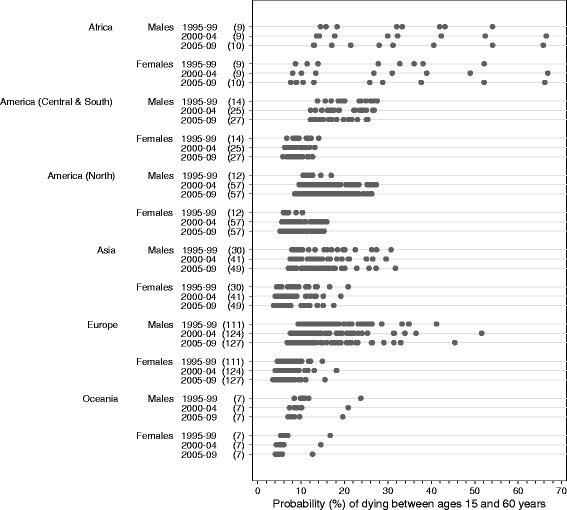
Probability of dying between ages 15 and 60: range, by continent, calendar period and sex. The numbers in brackets beside each calendar period denote the number of registries contributing life table data for that calendar period. Each dot on the graph represents a registry population or sub-population

**Fig. 4:**
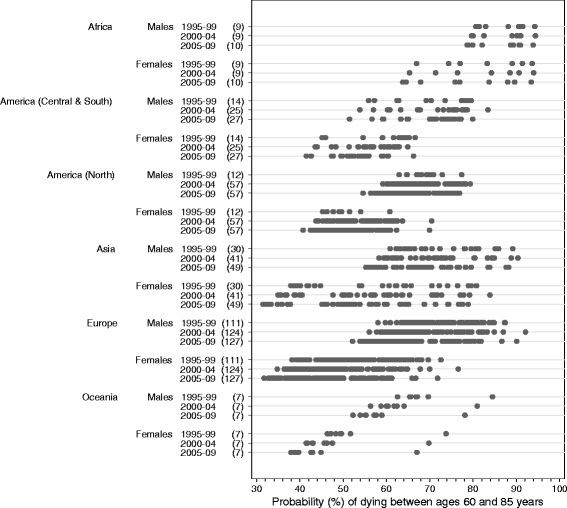
Probability of dying between ages 60 and 85: range, by continent, calendar period and sex. The numbers in brackets beside each calendar period denote the number of registries contributing life table data for that calendar period. Each dot on the graph represents a registry population or sub-population

**Fig. 5:**
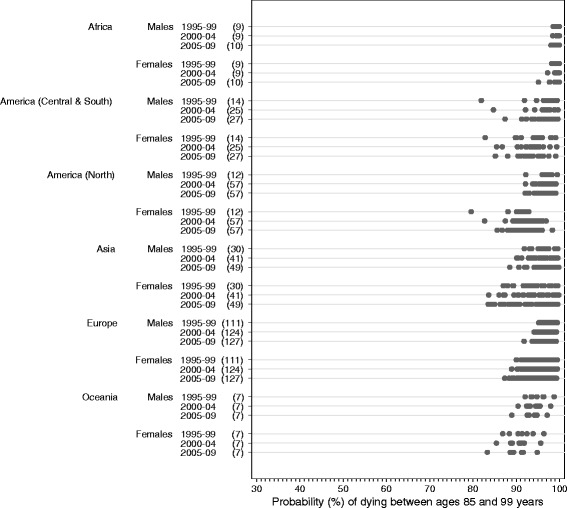
Probability of dying between ages 85 and 99: range, by continent, calendar period and sex. The numbers in brackets beside each calendar period denote the number of registries contributing life table data for that calendar period. Each dot on the graph represents a registry population or sub-population

These variations and trends in life expectancy at birth summarise the underlying patterns and trends in age-specific mortality, which also varied very widely (Table 3; Figs. 3, 4 and 5; Additional file 4). Worldwide, the greatest range in the probability of death among adults was seen in the age range 60 to 85 years, both during 1995–1999 (37.9% to 93.7% among females; 55.9% to 94.3% among males) and during 2005–2009 (31.5% to 93.5% among females; 51.4% to 93.9% among males).

**Fig. 6:**
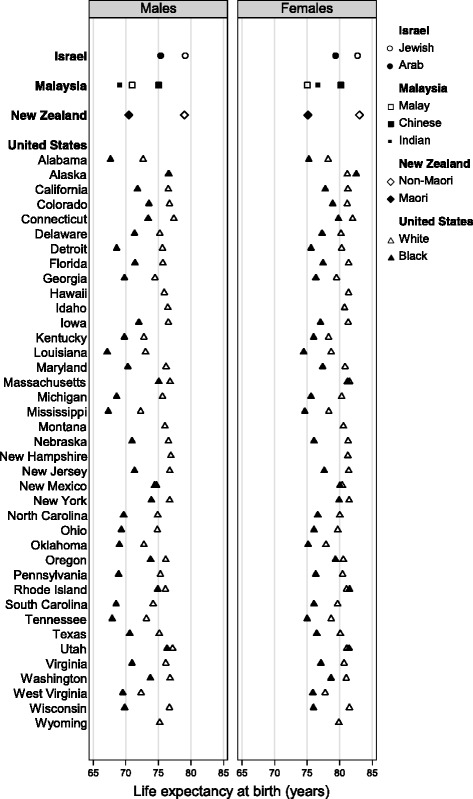
Life expectancy at birth (years) by race/ethnicity and sex: 2005–2009. Data are presented for Israel, Malaysia, New Zealand and 38* states of the United States. Hollow diamonds denote the majority ethnic group in each population. * Six metropolitan SEER registries were also included in the CONCORD-2 study, but the life tables used were those of the parent state (see text)

Where we obtained background mortality data by race or ethnic group, the majority group (whites in the United States, Jews in Israel, Non-Maoris in New Zealand) tended to have higher life expectancy at birth than the other subgroup(s). Malaysia (Penang Cancer Registry) was the exception, where life expectancy among the Chinese (23% of the population) was higher than among the majority Malay (50%) population [20] (Fig. 6; Additional file 4). Correspondingly, there were clear disparities in age-specific mortality, but the rates tended to converge among the elderly.

For some states in the US, the population of blacks is so small that death counts were not available for several age groups. We were therefore unable to construct robust life tables for blacks in Hawaii, New Hampshire, Montana, Idaho or Wyoming, even with the flexible Poisson model. For Utah and Alaska, the black life tables were also based on small counts, but data were available for enough age groups for us to construct life tables for use in survival analyses.

### Impact of using state- and race-specific life tables

We compared five-year net survival estimates for the 44 participating US registries for patients diagnosed during 2005–2009, obtained using state- and race-specific life tables that we had constructed using the flexible Poisson model, with the corresponding survival estimates derived with the national, race-specific life tables obtained from the National Center for Health Statistics (NCHS) [21]. For this comparison, we chose four cancers with very different prognosis: breast and prostate (high), colon (medium) and lung (low).

Absolute differences between the two sets of survival estimates were greatest for states where life expectancy at birth differed most from the national average, and for cancers with a better prognosis. They were smallest for states where life expectancy at birth differed least from the national average, and for cancers with a poor prognosis, where the majority of deaths were excess deaths. Differences were largest for men with prostate cancer, and for women with breast cancer, and smallest for lung cancer in both sexes (Table 4). The greatest difference was 3.6% for prostate cancer in Mississippi.

**Table 4:** Absolute differences in five-year net survival estimates obtained using national versus state-specific, race-specific life tables

	Absolute difference (%) in five-year net survival estimates			
	Colon	Lung	Breast	Prostate
**Alabama**	1.81	0.37	2.12	**3.30**
Alaska	–0.77	–0.10	–0.37	–1.10
California	–0.82	–0.17	–0.80	–1.16
Greater Bay Area	–0.84	–0.19	–0.87	–0.43
Greater California	–0.86	–0.15	–0.83	–1.37
Los Angeles	–0.73	–0.18	–0.65	–1.28
Colorado	–0.62	–0.18	–0.55	–0.80
Connecticut	–1.12	–0.33	–1.28	–1.24
Delaware	–0.07	–0.03	0.14	–0.17
Florida	–1.49	–0.45	–1.71	–0.72
Georgia	1.12	0.23	1.32	2.11
Atlanta	1.12	0.26	1.30	1.04
**Hawaii**	–2.27	–0.45	**–3.09**	–1.61
Idaho	–0.15	–0.09	–0.01	–0.45
Iowa	–0.21	–0.07	–0.50	–0.14
**Kentucky**	1.82	0.39	2.07	**3.32**
Louisiana	1.33	0.28	1.58	2.33
Maryland	–0.18	–0.03	–0.15	–0.20
Massachusetts	–0.42	–0.15	–0.60	–0.22
Michigan	0.38	0.08	0.42	0.57
Detroit	0.43	0.09	0.47	0.54
**Mississippi**	1.63	0.42	2.09	**3.56**
Montana	–0.19	–0.07	0.01	–0.59
Nebraska	–0.18	–0.08	–0.26	–0.31
New Hampshire	–0.24	–0.08	–0.26	0.04
New Jersey	–0.33	–0.11	–0.48	–0.24
New Mexico	–0.47	–0.09	–0.38	–1.12
New York	–0.85	–0.23	–1.02	–1.01
North Carolina	0.61	0.13	0.62	1.25
Ohio	0.82	0.19	0.93	1.19
Oklahoma	1.75	0.38	2.32	2.85
Oregon	–0.07	–0.03	0.05	–0.29
Pennsylvania	0.33	0.07	0.21	0.63
Rhode Island	–0.18	–0.07	–0.51	0.07
South Carolina	0.70	0.17	0.72	1.49
Tennessee	1.53	0.38	1.68	2.55
Texas	0.43	0.10	0.60	0.59
Utah	–0.67	–0.17	–0.17	–0.43
Virginia	0.24	0.02	0.28	0.42
Washington	–0.38	–0.08	–0.22	–0.49
Seattle	–0.37	–0.08	–0.20	–0.30
**West Virginia**	2.18	0.44	2.57	**3.51**
Wisconsin	–0.25	–0.10	–0.51	–0.24
Wyoming	0.10	0.02	0.54	–0.05

Absolute difference in the five-year net survival (%) estimate for all patients diagnosed during 2005–2009 in each US registry obtained with (a) national, race-specific life tables and (b) state- and race-specific life tables. A negative value indicates that the estimate obtained with state- and race-specific life tables was lower than the estimate obtained with national, race-specific life tables. Absolute differences greater than 3% are shown in bold, along with the associated registry name

## Discussion

In order to establish worldwide surveillance of population-based cancer survival trends in the CONCORD-2 study [1], we needed to obtain or construct life tables of background mortality by age, sex and calendar year that were as specific as possible for each registry population or sub-population.

This was particularly important in light of the tremendous intra-continental and even sub-national variations in background mortality. The UN Population Division, the World Health Organisation and the Global Burden of Disease study regularly produce life tables for countries worldwide [13, 16, 17, 22], but they are for countries, rather than sub-regions or ethnic/racial groups, and they may not accurately reflect the background mortality in the specific population(s) covered by a cancer registry.

We were obliged to use several methods to construct the life tables, because of the different types of data available from the registries (complete or abridged death and population counts; complete or abridged mortality rates; no reliable data). These methods involved different assumptions about the shape of age-mortality patterns and the rate of increase of mortality at older ages. The different assumptions made in the construction of the life tables may have had an impact on the subsequent estimates of net survival, and this warrants further investigation.

We recommend using the multivariable flexible Poisson model to construct life tables for future international comparisons of population-based cancer survival. We found that this method performed well, even for small populations. It does not rely on an external standard population or a pre-defined set of coefficients, and therefore does not make strong assumptions about the age-pattern of mortality. It was also recently found to perform better than the Elandt-Johnson method and a flexible relational method (based on the Ewbank approach) for small populations [8].

Life expectancy at birth varied by more than 30 years among the 327 populations examined in the 279 registries. In Canada alone, during 2005–2009, life expectancy differed by 10 years between residents of Nunavut (females 73.4 years; males 68.3 years) and British Columbia (females 83.4 years; males 78.9 years). These differences are probably explained by the very different demographic profiles of these two provinces: aboriginal people made up 86% of the population of Nunavut in 2011 [23].

In most populations, life expectancy increased during 1995–2009, but in Lesotho and South Africa it fell by as much as 6 years, most probably because of the HIV/AIDS epidemic emerging in those countries during the 1990s [24, 25].

We constructed ethnic or race-specific life tables for Israel, Malaysia (Penang Cancer Registry), New Zealand and the US. These life tables showed marked differences in background mortality between the ethnic and racial sub-populations in each country. In 5 of the 44 participating US states (Hawaii, New Hampshire, Montana, Idaho, Wyoming), it was not possible to construct sufficiently robust life tables for blacks. However, we were able to use race- or ethnic-specific life tables to estimate net survival in 39 of the 44 US registries, and for Israel, Penang (Malaysia) and New Zealand, controlling for background mortality by age and sex separately within each race or ethnicity. This was a strength of the CONCORD-2 study.

Examination of the impact of using race-specific life tables for each US state on estimates of net survival showed that age-standardised estimates differed by up to 3.6%, when compared with estimates obtained with the national race-specific life tables that have been used in the past. The differences were more marked for cancers with better prognosis. This is in line with previous findings [2, 26]. The largest difference observed was in the estimate of age-standardised five-year net survival for prostate cancer in Mississippi, which was 3.6% higher when derived with state- and race-specific life tables than when using national life tables. The explanation is that background mortality among adults in Mississippi is considerably higher than the US national average, for both blacks and whites. National life tables therefore under-estimate background mortality in Mississippi, leading us to over-estimate excess mortality and subsequently underestimate net survival. Of note, we did not investigate age-, sex- or race-specific differences in net survival estimated with the alternative life tables. Differences in net survival from those obtained with national life tables will be larger in some of the groups defined by age and race and, for other cancers, sex. This is a further reason for using the most specific life tables that can be obtained.

Stroup et al. [26] recently examined the differences between estimates of relative survival by age, sex and race for 17 SEER registries, obtained either with state- and race-specific life tables or with national, race-specific life tables. The differences were greatest for patients aged 85 years or over, and differed in both direction and magnitude by race and sex. They deemed the NCHS state- and race-specific life tables unreliable above age 85 and recommended against using them to estimate relative survival for patients aged 85 and older. We have compared the probabilities of dying between ages 85 and 99 years for each state, race and sex derived from the CONCORD-2 life tables with those derived from the national, race-specific life tables available from NCHS. Estimates of the probability of dying between ages 85 and 99 were higher for black males and females, and to a lesser extent for white males, when derived from the CONCORD-2 state- and race-specific life tables than when derived with the national life tables. For white females, the estimates derived from the CONCORD-2 life tables were fairly evenly distributed around the corresponding national estimates.

We opted to use life tables that were specific to each registry or sub-population, wherever possible, in order to reflect as closely as possible the background mortality that an individual cancer patient would be expected to experience. This is critical when estimating net survival. Accurate life tables allow us to estimate the net survival of cancer patients within each population, rather than an approximation obtained with a less specific life table. This is particularly important for worldwide comparisons of cancer survival.

This was the first opportunity for us to use our flexible Poisson model [8] to construct life tables for such a large and disparate set of populations. The model generally performed well, even when the death counts were sparse. In some US states (California, New York, Alaska and Colorado), however, where the races classified as “Other” (American Indian/Alaska Native, Asian/Pacific Islander) made up a large proportion of the total population, the fit of the life table for blacks was not ideal, particularly at the oldest ages. This is probably because the interaction between race and age was modelled as a continuous variable, meaning that greater weight was given to the shape of the mortality function for “other” races at older ages than to that for blacks. This may have contributed to some over-estimation of background mortality among elderly blacks. We determined that inclusion of an interaction between race and age in the model as dummy variables (see Additional file 5) provided a better fit of the race-specific mortality rates to the underlying data, and we produced a new set of life tables for the US registries with this approach. We have also explored the inclusion of calendar year as a covariable: we believe this could facilitate construction of more robust life tables for future comparisons of cancer survival.

In some circumstances, we were limited by the availability of data, in others by data quality. For some populations, raw infant and child mortality rates were very low, suggesting some undercounting of infant deaths. In some populations, (sometimes the same populations), mortality rates at very old ages were remarkably high, perhaps indicating inaccurate reporting of the age at death (too high) or undercounting of older-age populations. Where possible, we compared our estimates of life expectancy with those available from the national statistical authority for the country or region covered by the cancer registry. These were generally very comparable. The quality and completeness of civil and vital registration statistics varies substantially around the world, however, and only about one-third of all deaths are actually registered. To improve the robustness of research on demographic trends it is critical to push for the improvement of vital statistics data worldwide [27, 28]. This would benefit international public health research, including international comparisons of cancer survival, by enabling wider participation, particularly in low- and middle-income countries.

## Conclusions

The methods by which life tables are constructed for the estimation of cancer survival depend on the nature and detail of the available data on death and population counts, or mortality rates.

To our knowledge, this study represents the first time such a large number of national and sub-national life tables have been constructed on a global scale. We found wide variations and major changes over time in life expectancy at birth, and in patterns of mortality by age, between 327 populations in the territory of 279 participating registries. This highlights the importance of using life tables that are as specific as possible to the populations or sub-populations for which comparisons of net survival are required, in order to control adequately for variations in background mortality between populations and over time.

The life tables used by the CONCORD programme are all available online. They can be downloaded from the Cancer Survival Group website [29].

## Additional files

Additional file 1: Additional information on the selection of knot locations for the flexible Poisson model. (DOCX 18 kb)
Additional file 2: Model for constructing race/ethnic-specific life tables using a continuous interaction term between race and age. (DOCX 13 kb)
Additional file 3: Example of a life table report. (PDF 38 kb)
Additional file 4: Background mortality in 327 populations covered by 279 registries during 1995–2009: mean life expectancy at birth (years), infant mortality rate per 1,000 live births, childhood mortality rate per 1,000 live births, and probabilities (%) of dying between exact ages 15 and 60 years, 60 and 85 years, and 85 and 99 years, by sex and calendar period. (XLS 386 kb)
Additional file 5: Alternative model for constructing race/ethnic-specific life tables using dummy interaction terms. (DOCX 16 kb)

## Abbreviations

NCHS: National center for health statistics
SEER: Surveillance, epidemiology, and end results program
UNPD: United nations population division
