# Supplementary material for: Life tables for global surveillance of cancer survival (the CONCORD programme): data sources and methods
Source: BMC Cancer. 2017 Feb 27;17:159. doi: 10.1186/s12885-017-3117-8 (PMC5327577; doi:10.1186/s12885-017-3117-8)
Supplement: Additional file 1: — Additional information on the selection of knot locations for the flexible Poisson model. (DOCX 18 kb) [file 12885_2017_3117_MOESM1_ESM.docx]

**Additional file 1: Additional information on the selection of knot locations for the flexible Poisson model**

**Splines are flexible functions made up of piecewise polynomials. They are smoothly joined together at locations called knots. For all populations, knot locations were specified *a priori* at ages 0 and 1 and at the median age at death in the oldest age group**^[[1]](#footnote-1)^**. For death and population counts by single year of age, an additional knot was also specified at age 2.**

**For all populations, an additional 3 to 5 knots between ages 3 and 50 were determined through iterations.** Mortality tends to increase log-linearly from around age 50 onwards. As such, only one knot (at the median age at death in the oldest age group) was specified above age 50 in the model. Random locations for the 3 to 5 additional knots were simulated 100 times for each of five knot patterns. The resulting models were ranked according to **Akaike Information Criterion (AIC) values. Patterns of knot locations were selected by evaluation of AIC values, checking for recurring knot patterns within the models with the lowest AIC values. This process is described in greater detail elsewhere [**[**8**](#_ENREF_8)**].**

**For registry populations where death and population counts were more sparse, this process did not always yield satisfactory results. In such cases, we used knowledge of where we would expect changes or inflections in the shape of the mortality curve, based on the pattern of raw data points and recurring knot locations for other registry populations, or for that same population but for different calendar years.**

1. **The oldest age group was defined differently depending on whether the death and population counts were available by age group (“abridged”: 100 registries) or single year of age (“complete”: 72 registries). Where death and population counts were abridged, the oldest age group was typically defined as age 85 years and over. Where death and population counts were complete, we grouped information on death and population counts for ages 90 years and above.**  [↑](#footnote-ref-1)
