# Supplementary material for: Life tables for global surveillance of cancer survival (the CONCORD programme): data sources and methods
Source: BMC Cancer. 2017 Feb 27;17:159. doi: 10.1186/s12885-017-3117-8 (PMC5327577; doi:10.1186/s12885-017-3117-8)
Supplement: Additional file 2: — Model for constructing race/ethnic-specific life tables using a continuous interaction term between race and age. (DOCX 13 kb) [file 12885_2017_3117_MOESM2_ESM.docx]

**Additional file 2: Model for constructing race/ethnic-specific life tables using a continuous interaction term between race and age**

$$log\left( d_{x,i} \right)=\beta_{0}+f\left( x \right)+\sum_{i=2}^{n} {\beta_{i}race}_{i}+g\left( x\_race \right)+log\left( {pyrs}_{x,i} \right)$$

where $x$ denotes age in years, $i$ denotes race/ethnicity, $d_{x,i}$ denotes the age- and race/ethnic-specific counts of deaths in the population, $\beta_{0}$ denotes the coefficient at baseline (i.e. the log of the mortality rate at the reference age for the reference race/ethnic group), $f(x)$ denotes a restricted cubic spline function on age, $g(x\_race)$ denotes a restricted cubic spline function on the continuous interaction between race and age, $\beta_{i}$is the coefficient for the main effect of ${race}_{i}$ (i.e. how mortality differs at the reference age in race/ethnic group $i$ compared to the reference race/ethnic group), and ${pyrs}_{x,i}$ denotes the age- and race/ethnic-specific person-years at risk in the population.

In addition to the knots on age specified in Additional file 1, three knots were initially specified for the continuous interaction between race/ethnicity and age, at the 25^th^, 50^th^ and 75^th^ percentiles of their distributions. An algorithm embedded in *mvrs* [[9](#_ENREF_9)] and based on the Akaike Information Criterion, then enabled us to identify the number of knots to be included in the final model.
