# Supplementary material for: Life tables for global surveillance of cancer survival (the CONCORD programme): data sources and methods
Source: BMC Cancer. 2017 Feb 27;17:159. doi: 10.1186/s12885-017-3117-8 (PMC5327577; doi:10.1186/s12885-017-3117-8)

CONCORD-2 life tables: Denmark **10 February 2015**

**Method:**	Life tables for Denmark for the years 1995 and 2012 were constructed from the numbers of deaths and population counts by single year of age, sex and calendar year, using Poisson regression and flexible functions. Life tables for intervening years were constructed by linear interpolation. See article in *The Lancet* (http://dx.doi.org/10.1016/S0140-6736(14)62038-9) for more details.
**Source:**	Statistics Denmark (Danmarks Statistik)
**Registry population (2009):**	5,524,430

Year 1995

Table 1: Summary statistics

Sex	Life expectancy at birth (years)	Probability (%) of dying between exact ages		
		15-60 years	60-85 years	85-99 years
**Male**	72.8	14.7	77.1	98.5
**Female**	78.1	9.3	60.2	95.0

Figure 1: Observed and fitted mortality rates (mx; logarithmic scale) by age and sex

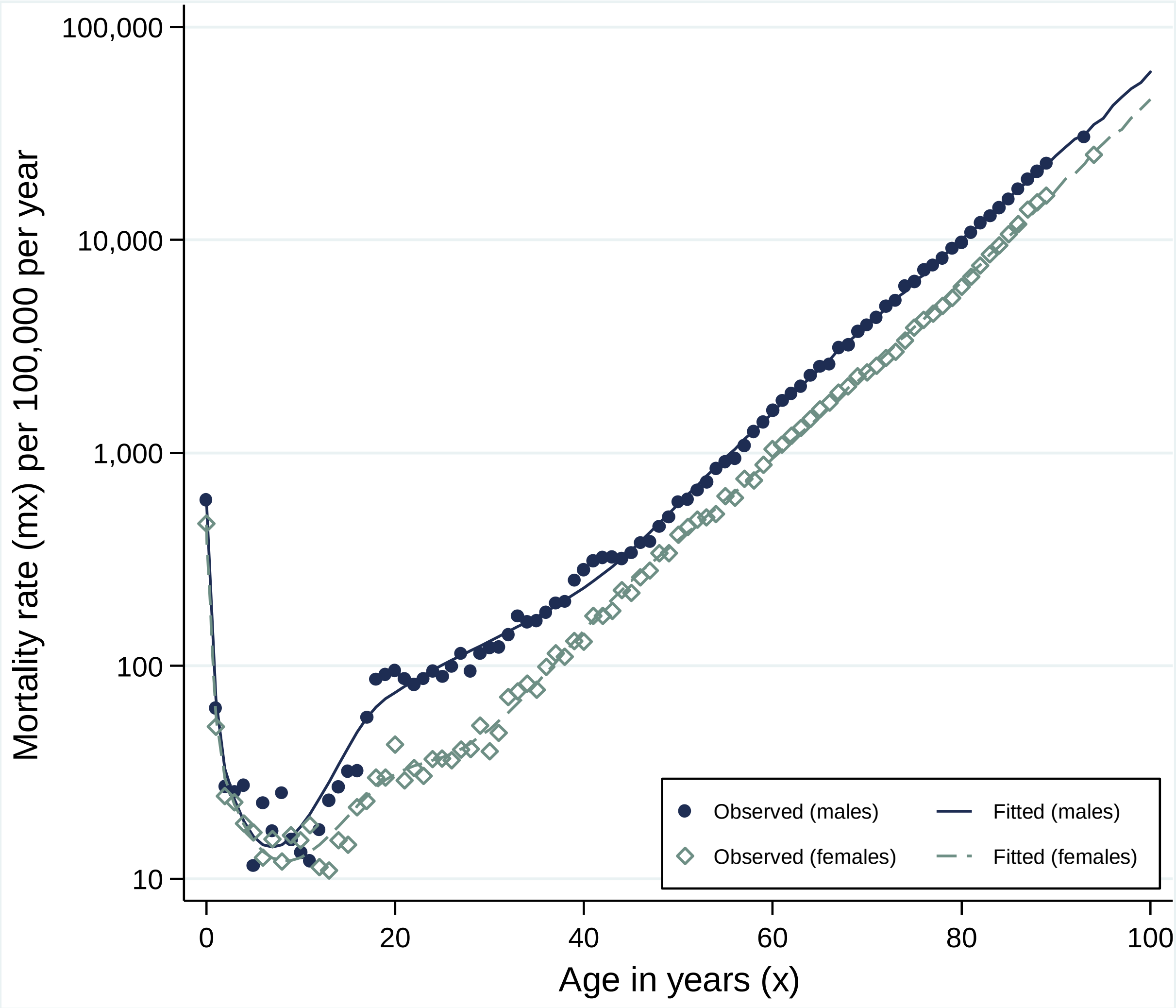

Figure 2: Observed and fitted mortality rates (mx; arithmetic scale) by broad age band and sex

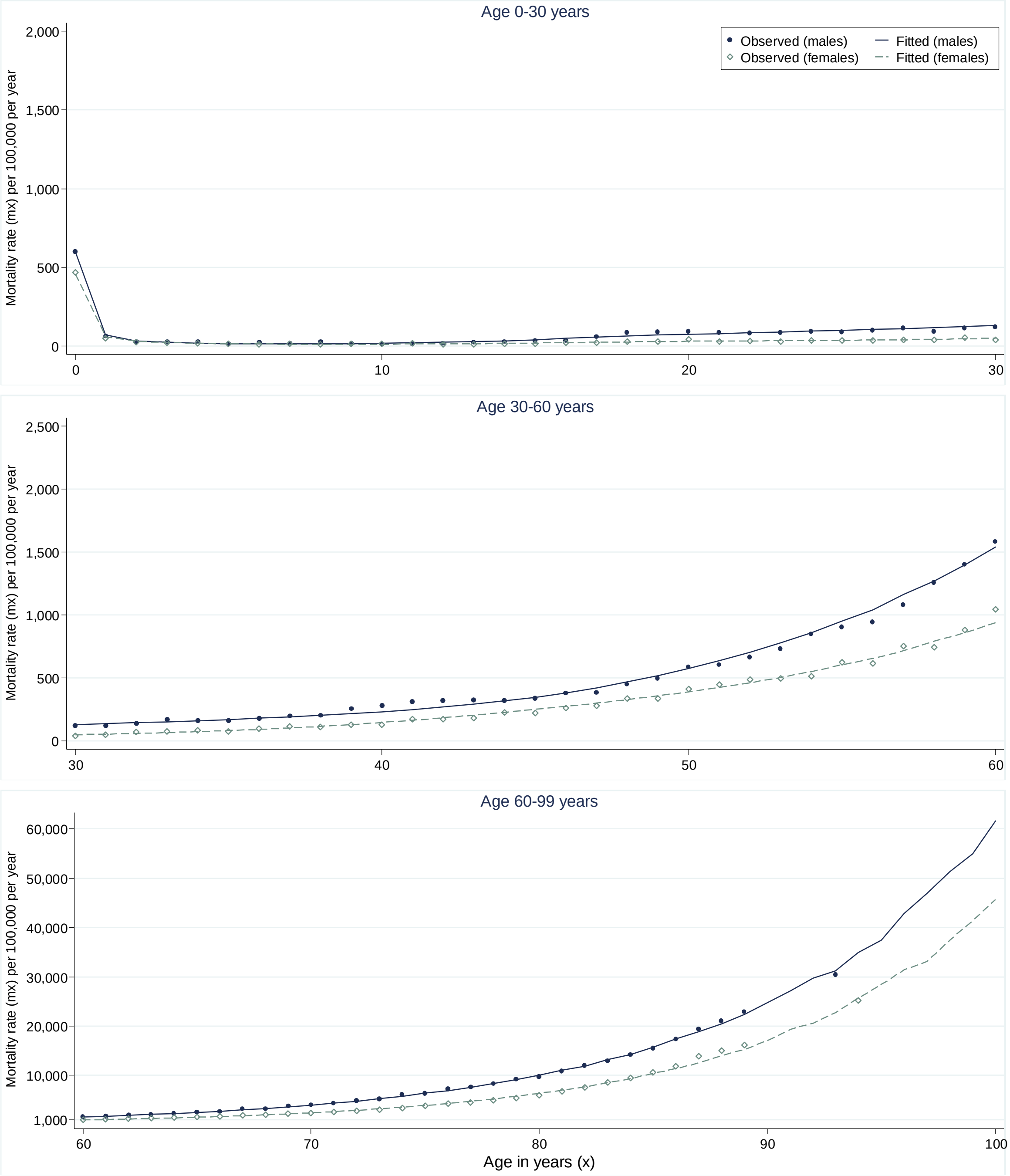

Figure 3: Fitted versus observed mortality rates: residual deviance

We plotted the deviance residuals of the model against age to assess goodness of fit. Deviance residuals are a measure of how closely the modelled values fit the observed data. They should be approximately normally distributed if the model fits the data well. The ideal range is between -2 and +2 (the red lines).

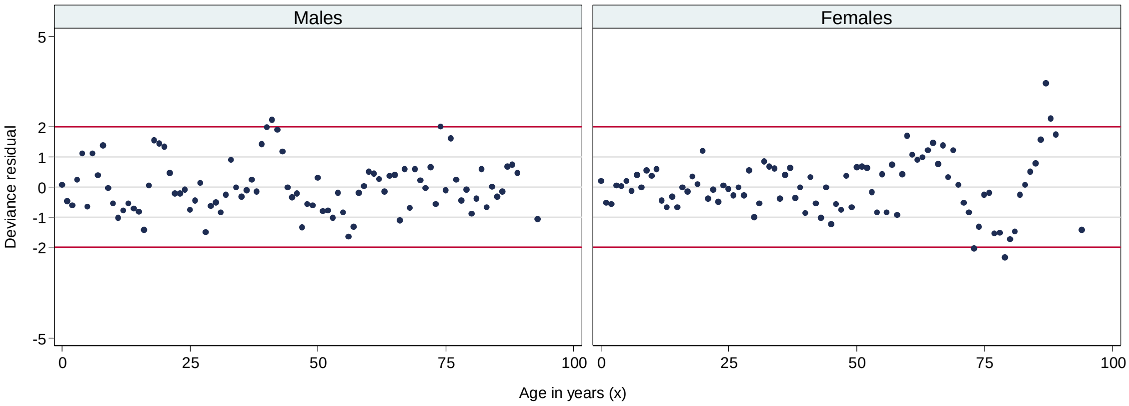

Table 1: Summary statistics

Sex	Life expectancy at birth (years)	Probability (%) of dying between exact ages		
		15-60 years	60-85 years	85-99 years
**Male**	77.9	9.0	62.5	97.3
**Female**	82.0	5.5	48.5	92.5

Figure 1: Observed and fitted mortality rates (mx; logarithmic scale) by age and sex

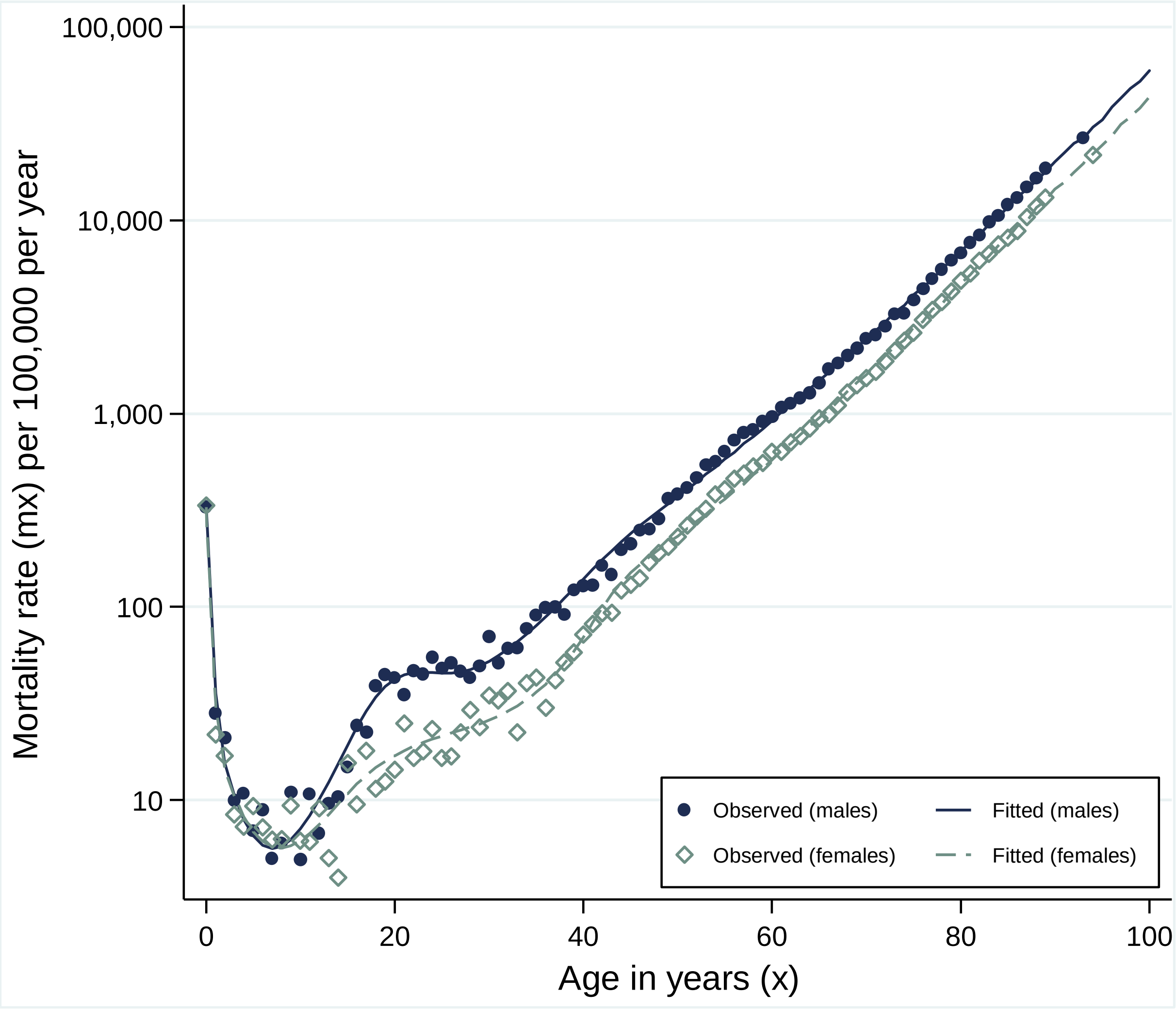

Figure 2: Observed and fitted mortality rates (mx; arithmetic scale) by broad age band and sex

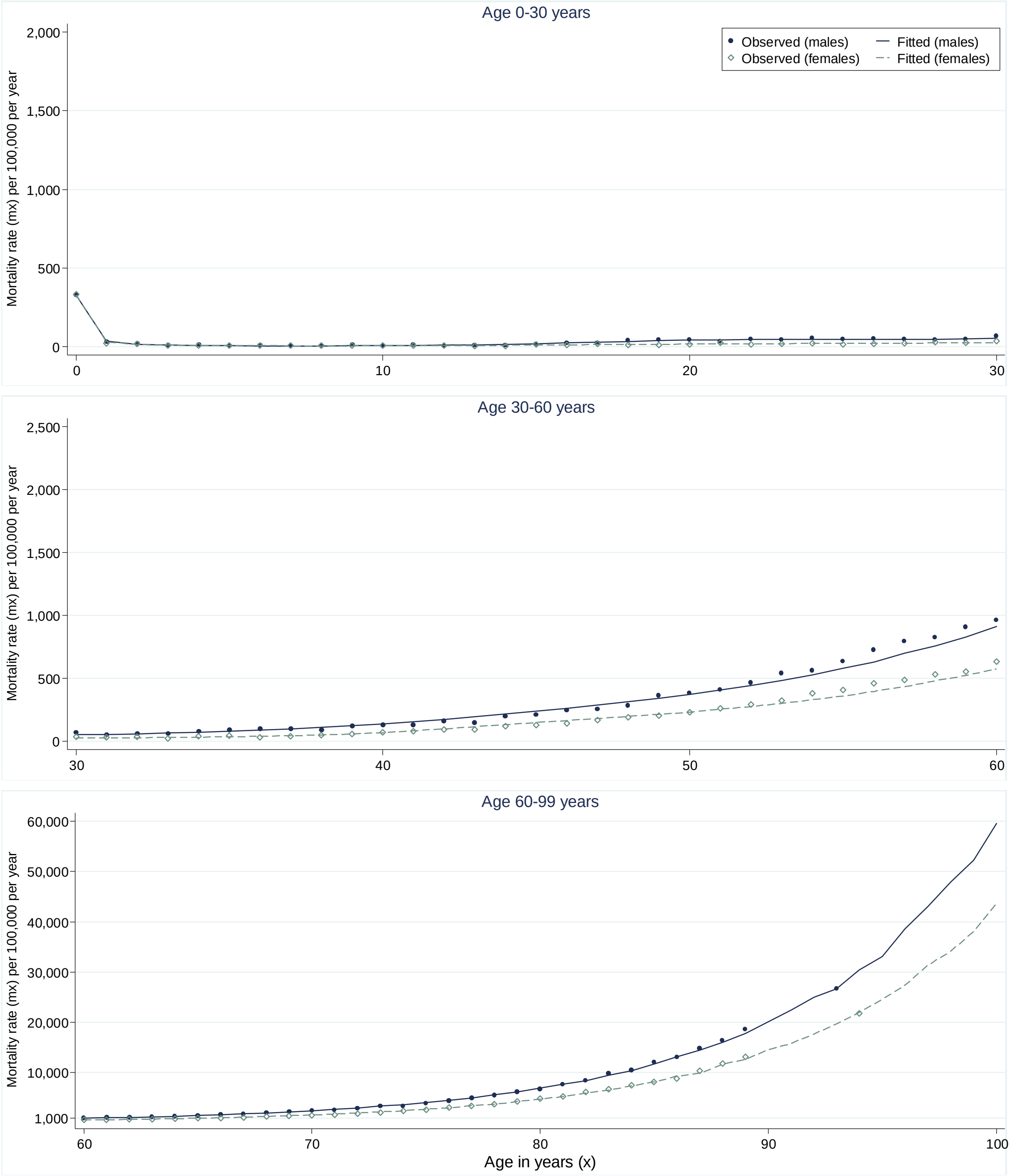

Figure 3: Fitted versus observed mortality rates: residual deviance

We plotted the deviance residuals of the model against age to assess goodness of fit. Deviance residuals are a measure of how closely the modelled values fit the observed data. They should be approximately normally distributed if the model fits the data well. The ideal range is between -2 and +2 (the red lines).

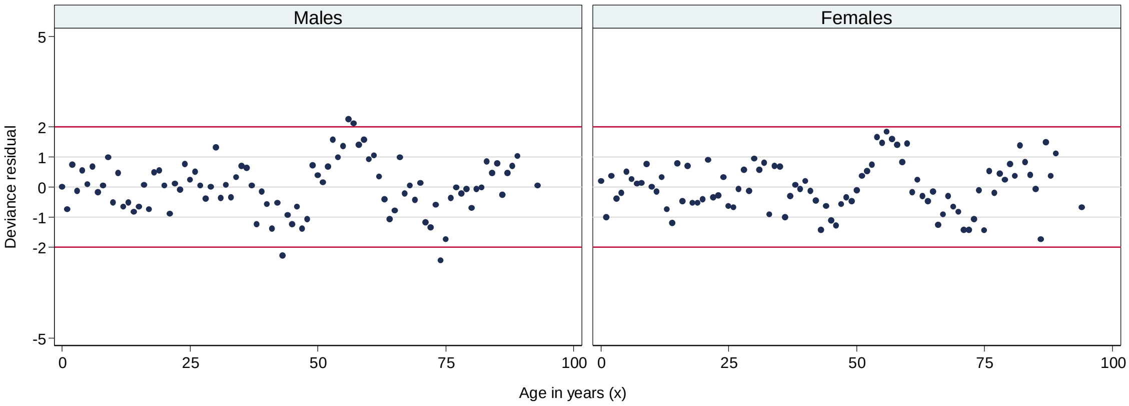

Supplement: Additional file 3: — Example of a life table report. (PDF 38 kb) [file 12885_2017_3117_MOESM3_ESM.pdf]
