# Supplementary material for: Life tables for global surveillance of cancer survival (the CONCORD programme): data sources and methods
Source: BMC Cancer. 2017 Feb 27;17:159. doi: 10.1186/s12885-017-3117-8 (PMC5327577; doi:10.1186/s12885-017-3117-8)
Supplement: Additional file 5: — Alternative model for constructing race/ethnic-specific life tables using dummy interaction terms. (DOCX 16 kb) [file 12885_2017_3117_MOESM5_ESM.docx]

**Additional file 5: Alternative model for constructing race/ethnic-specific life tables using dummy interaction terms**

$$log\left( d_{x,i} \right)=\beta_{0}+f\left( x \right)+\sum_{i=2}^{n} \left( {\beta_{i}race}_{i}+g\left( {x\_race}_{i} \right) \right)+log\left( {pyrs}_{x,i} \right)$$

where $x$ denotes age in years, $i$ denotes race/ethnicity, $d_{x,i}$ denotes the age- and race/ethnic-specific counts of deaths in the population, $\beta_{0}$ denotes the coefficient at baseline (i.e. the log of the mortality rate at the reference age for the reference race/ethnic group), $f(x)$ denotes a cubic spline function on age, $g(x\_race_{i})$ denotes a cubic spline function on the dummy interaction between $race_{i}$ and age, $\beta_{i}$denotes the main effect of ${race}_{i}$ (i.e. how mortality differs at the reference age in race/ethnic group $i$ compared to the reference race/ethnic group, and ${pyrs}_{x,i}$ denotes the age- and race/ethnic-specific person-years at risk in the population.

In addition to the knots on age specified in Additional file 1, four knots were initially specified for the interaction between each race/ethnicity and age, at the 25^th^, 50^th^ and 75^th^ percentiles of their distributions. An algorithm embedded in *mvrs* and based on AIC then identified the number of knots to be included in the final model for each.
